# A Rare Cause of Small Bowel Obstruction: Malignant Metastatic Sarcoma

**DOI:** 10.7759/cureus.106126

**Published:** 2026-03-30

**Authors:** Alexander Ponce, Tammy Davis, Thomas M Jones

**Affiliations:** 1 General Surgery, Central Surgical Associates, Jackson, USA; 2 Internal Medicine, Mississippi Baptist Medical Center Jackson, Jackson, USA; 3 Internal Medicine, William Carey University College of Osteopathic Medicine, Hattiesburg, USA; 4 Surgery, Central Surgical Associates, Jackson, USA

**Keywords:** laparotomy, malignancy, sarcoma, sbo, small bowel obstruction, surgery

## Abstract

Small bowel obstruction is a common reason for hospital admission and is most frequently caused by postoperative adhesions. Malignancy is a less common etiology and typically results from a focal obstructing lesion. We present the case of a 67-year-old female who developed small bowel obstruction due to diffuse sarcomatous implants throughout the abdomen and pelvis. Computed tomography demonstrated dilated small bowel with a pelvic transition point. After failure of conservative management, the patient underwent surgical exploration, which revealed hundreds of small malignant lesions involving the small bowel, omentum, and peritoneum, resulting in bowel fixation and obstruction. The obstructed segment of small bowel was resected, and a diverting loop colostomy was created because of impending obstruction at the affected rectum. Pathology revealed a high-grade, poorly differentiated sarcomatous malignancy. This case highlights a rare malignant cause of small bowel obstruction and emphasizes the importance of considering underlying malignancy in patients who fail non-operative management, particularly those with prior oncologic risk factors.

## Introduction

Small bowel obstruction (SBO) is one of the most common surgical presentations encountered in the hospital setting [1]. The majority of cases are caused by postoperative adhesions [2], followed by hernias and inflammatory conditions [1,2]. Malignancy represents a less common etiology of small bowel obstruction (SBO) but is an important consideration, particularly in patients with a history of cancer or failure of conservative management [2,3].

Malignant small bowel obstruction most frequently results from extrinsic compression, intraluminal mass effect, or peritoneal carcinomatosis [4]. Colorectal cancer is a common cause of malignant bowel obstruction overall [5]; however, other neoplastic processes involving the gastrointestinal tract, including gastrointestinal stromal tumors, carcinoid tumors, and metastatic disease, have also been reported [1,5]. The clinical presentation and operative findings of malignant SBO vary widely depending on tumor type, disease burden, and pattern of spread [5,6]. Diffuse peritoneal involvement from sarcomatous malignancy causing mechanical small bowel obstruction is particularly rare, especially in the absence of a dominant mass lesion [6].

Here, we present a rare case of a 67-year-old female who presented with small bowel obstruction and was found on exploratory laparotomy to have extensive, diffuse sarcomatous implants involving the omentum and small bowel, with hundreds of lesions causing mechanical obstruction. The patient’s history of prior pelvic radiation is notable, as radiation exposure is a known risk factor for secondary soft-tissue sarcomas, although a definitive causal relationship cannot be established in this case. This case highlights an unusual malignant etiology of SBO and underscores the importance of considering diffuse intra-abdominal malignancy in patients who fail non-operative management [4,6].

## Case presentation

**Figure 1:**
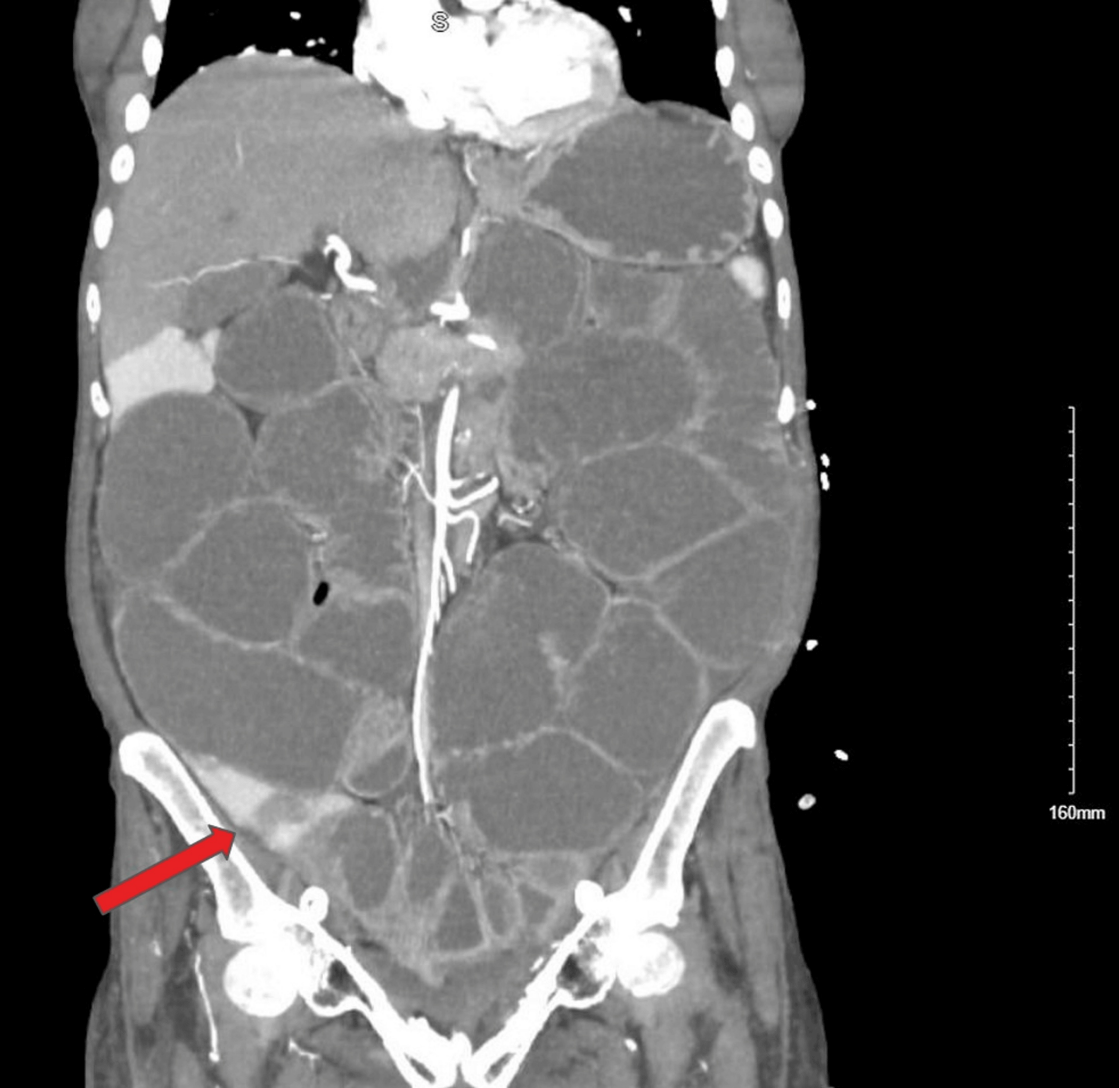
Computed tomography angiography of the abdomen and pelvis demonstrating dilated loops of small bowel with a transition point in the pelvis (arrow), consistent with mechanical small bowel obstruction. The transition point represents the site of obstruction where the proximal bowel is dilated and the distal bowel is decompressed.

A 67-year-old female with a past medical history significant for prior cervical cancer, end-stage renal disease, severe malnutrition, hypertension, and deep vein thrombosis status post inferior vena cava filter presented to the emergency department with abdominal pain, melena, and symptoms concerning for small bowel obstruction. Her cervical cancer had been treated approximately 20 years ago with a hysterectomy followed by radiation for concerns of nodal spread. Initial laboratory studies demonstrated leukocytosis, anemia, and elevated lactate. Computed tomography angiography of the abdomen and pelvis demonstrated small bowel obstruction with a transition point in the pelvis, bilateral renal cortical atrophy, and a left percutaneous nephrostomy tube (Figure 1). The patient reported a recent hospitalization within the prior month for a duodenal ulcer treated with endoscopic clipping.

**Figure 2:**
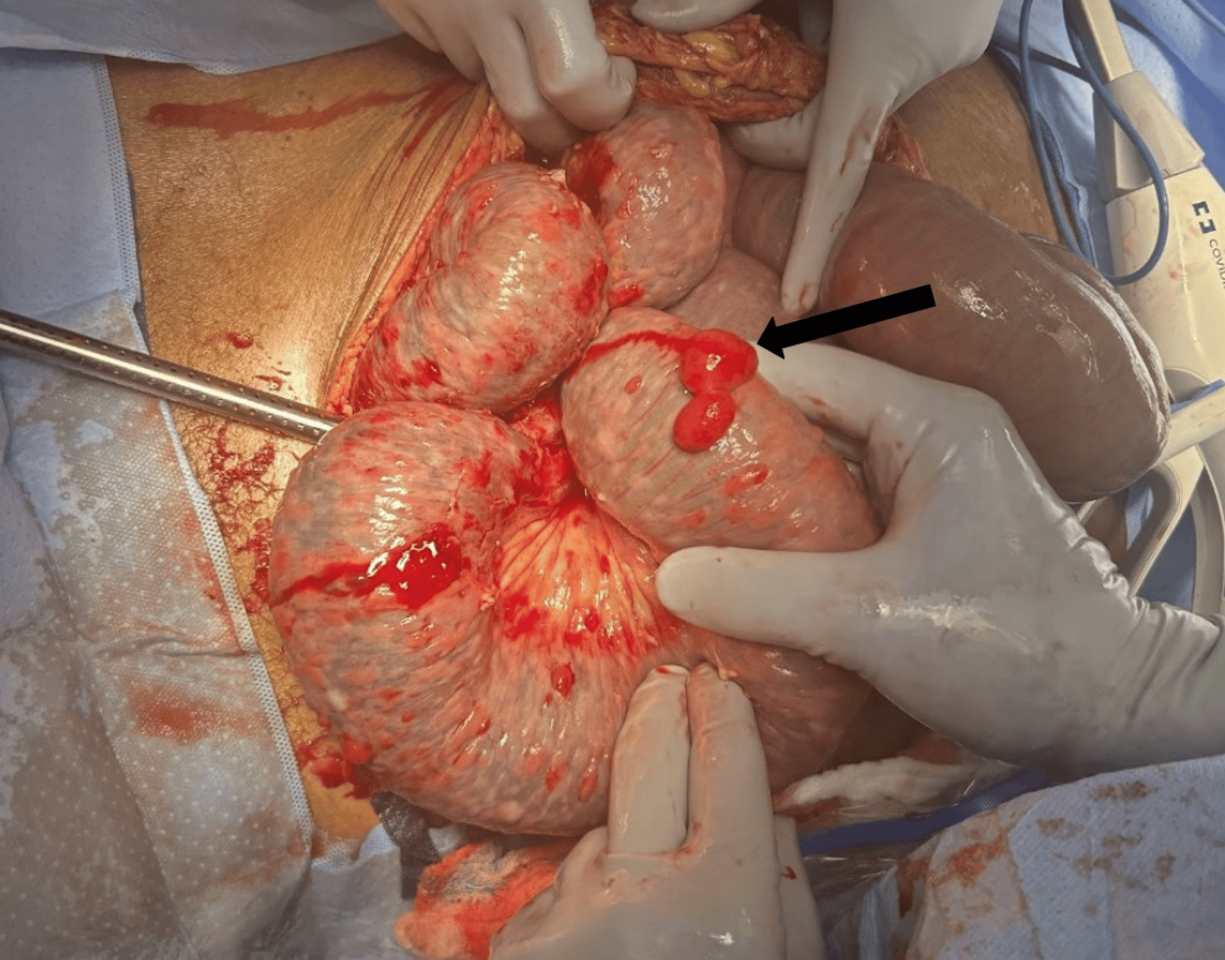
Intraoperative photograph demonstrating diffuse tumor implants along the serosal surface of the small bowel (arrow), which caused bowel fixation and mechanical small bowel obstruction.

Given concern for upper gastrointestinal bleeding, esophagogastroduodenoscopy was attempted on hospital day one but was aborted due to severe esophagitis and hemodynamic instability. General surgery was consulted for management of the small bowel obstruction, and the patient was initially managed non-operatively with nasogastric decompression, bowel rest, and serial abdominal examinations. After four days of persistent distention despite intermittent bowel movements, the patient underwent exploratory laparotomy for failure of non-operative management. Intraoperatively, the small bowel was markedly distended, with the omentum and loops of bowel adherent within the pelvis. Innumerable small, friable, granulomatous-appearing tumors (Figure 2) were identified diffusely involving the omentum, the distal small bowel, and the rectosigmoid junction. Frozen section analysis suggested spindle-cell neoplasms from multiple sampled sites. The neoplastic lesions caused kinking and fixation of the bowel at the pelvic transition point, resulting in mechanical obstruction.

**Figure 3:**
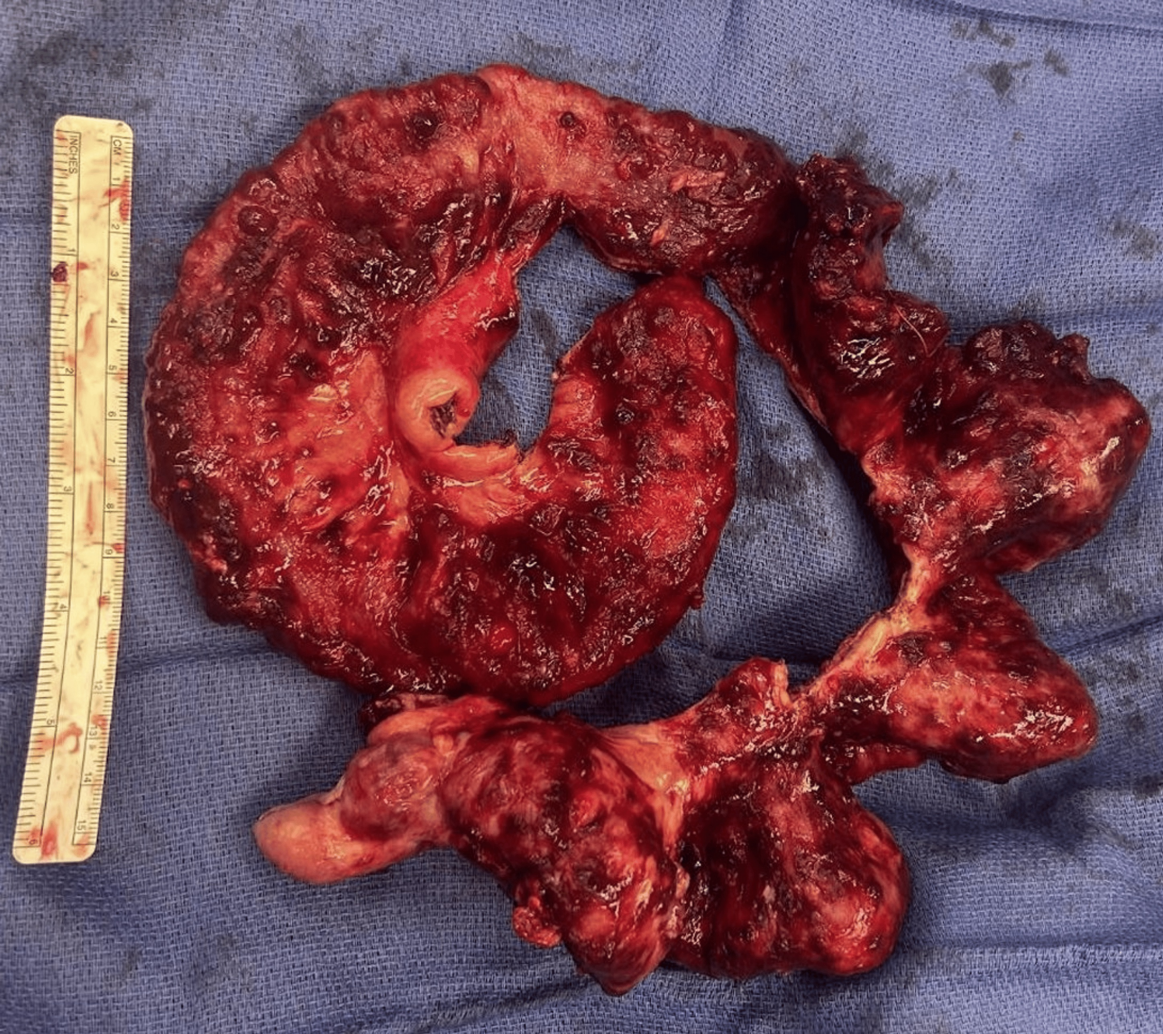
Gross specimen of resected small bowel demonstrating areas of tumor involvement and nonviable bowel. Resection was performed due to obstruction and compromised bowel viability caused by diffuse sarcomatous involvement.

Given the diffuse, extensive nature of the disease and stricturing, likely a sequela of radiation therapy, there was concern for recurrent obstruction at the rectum. A loop sigmoid colostomy was brought up for diversion, and nonviable small bowel (Figure 3) was resected. Specimens were sent for definitive pathological analysis, and the abdomen was closed in standard fashion. Postoperatively, the patient was managed with bowel rest and total parenteral nutrition, followed by gradual advancement of diet as tolerated. The patient was ultimately transferred to a long-term acute care facility after stabilization. Final pathology demonstrated a high-grade, poorly differentiated sarcomatous malignancy.

Clinical timeline

The patient presented with abdominal pain, melena, and symptoms concerning for small bowel obstruction. Imaging demonstrated a small bowel obstruction with a transition point in the pelvis. The patient was initially managed non-operatively with nasogastric decompression, bowel rest, and serial abdominal examinations. After four days of persistent abdominal distention despite conservative management, the patient underwent exploratory laparotomy, which revealed diffuse sarcomatous implants involving the omentum and small bowel, causing mechanical obstruction. The obstructed segment of small bowel was resected, and a diverting loop colostomy was created. Final pathology demonstrated a high-grade, poorly differentiated sarcomatous malignancy, and the patient was subsequently transferred to a long-term acute care facility after stabilization.

## Discussion

Malignant small bowel obstruction most commonly results from a single obstructing intraluminal mass, extrinsic compression, or adhesive disease in patients with prior abdominal surgery [4]. Diffuse peritoneal involvement leading to obstruction is less frequently encountered, particularly in the absence of a dominant mass lesion [6]. The differential diagnosis for this patient’s presentation initially included common causes of small bowel obstruction, such as postoperative adhesions, as well as malignant etiologies, including recurrent or metastatic cervical malignancy, peritoneal carcinomatosis, primary gastrointestinal malignancy such as gastrointestinal stromal tumor, and radiation-related stricturing disease. However, the presence of diffuse serosal tumor implants identified intraoperatively and the final pathology demonstrating a high-grade, poorly differentiated sarcomatous malignancy made these alternative diagnoses less likely. The extent and multiplicity of neoplastic implants observed in this patient were unusual and contributed to mechanical kinking, fixation, and restricted bowel mobility at the pelvic transition point, ultimately leading to failure of non-operative management and necessitating surgical intervention.

Among primary small bowel malignancies, gastrointestinal stromal tumors (GISTs) represent the most common mesenchymal (sarcomatous) tumors of the gastrointestinal tract and are a recognized malignant cause of small bowel obstruction [7]. GISTs typically demonstrate spindle-cell morphology on histology [8] and account for approximately 1-3% of all primary gastrointestinal malignancies [9], with 20-30% arising in the small intestine [8]. Although obstruction is an uncommon presenting feature, GIST remains among the dominant histologic diagnoses when primary small bowel neoplasms result in malignant obstruction [10]. The diffuse intra-abdominal distribution and absence of a dominant primary mass in this case, however, are not consistent with a typical presentation of GIST. Final pathology demonstrated a high-grade, poorly differentiated sarcomatous malignancy. Due to the poorly differentiated nature of the tumor and limited available records, definitive classification of the sarcoma subtype and primary site of origin could not be established. Therefore, this case is best classified as a diffuse intra-abdominal sarcomatous malignancy of uncertain primary origin causing mechanical small bowel obstruction.

Several factors may have contributed to the development of sarcoma in this patient. Prior therapeutic radiation exposure is a well-established risk factor for secondary soft-tissue sarcoma [11]. Large cancer registry analyses have demonstrated that patients treated with radiotherapy have a significantly increased long-term risk of developing soft-tissue sarcoma, with standardized incidence ratios reaching 2.20 more than 15 years after exposure compared with 1.21 in non-irradiated patients [11]. This patient’s history of pelvic chemoradiotherapy for cervical cancer more than 20 years prior represents a potential risk factor and is consistent with the prolonged latency period described in radiation-associated sarcomagenesis [12]. In addition, certain classes of chemotherapeutic agents, particularly alkylating agents, have independently been associated with an increased risk of secondary malignancies, including sarcomas [13]. Radiation-induced sarcomas remain rare, accounting for approximately 3-6% of all sarcoma diagnoses [14], with a cumulative incidence of 0.03-0.2% at 10 years following radiotherapy [15].

Beyond oncogenesis, prior radiation exposure may also have contributed to the extensive adhesive disease observed intraoperatively. Pelvic radiation is known to cause chronic peritoneal inflammation, microvascular injury, and fibrosis, resulting in mesothelial damage and dysregulated wound healing [16]. These changes promote fibrin deposition and impaired fibrinolysis, predisposing patients to adhesion formation [17]. In the setting of diffuse sarcomatous peritoneal involvement, tumor-associated inflammation may further exacerbate this response, leading to dense adhesions, bowel fixation, and subsequent mechanical obstruction [18]. However, a definitive causal relationship between prior radiation exposure and the development of sarcoma in this patient cannot be established based on the available pathological findings.

This case highlights the importance of maintaining a high index of suspicion for malignant etiologies in patients with small bowel obstruction who fail conservative management, particularly in those with a history of prior malignancy, radiation exposure, or unexplained malnutrition [5,8]. In this patient, diffuse intra-abdominal sarcomatous involvement was not clearly identified on preoperative imaging, and a definitive diagnosis was only established at the time of surgical exploration [6,15].

This case report has several limitations. Complete outside medical records, prior imaging, detailed laboratory trends, and additional oncologic records were not available for review at the time of manuscript preparation, which limited further characterization of the patient’s disease course and oncologic history. In addition, the poorly differentiated nature of the tumor limited definitive classification of the sarcoma subtype and determination of the primary site of origin. Despite these limitations, intraoperative findings and pathology confirmed diffuse intra-abdominal sarcomatous involvement resulting in mechanical small bowel obstruction.

## Conclusions

This case demonstrates a rare cause of small bowel obstruction due to diffuse intra-abdominal involvement from a high-grade, poorly differentiated sarcomatous malignancy of uncertain primary origin, in contrast to the more common focal malignant etiologies. It highlights the importance of maintaining a high index of suspicion for malignant causes of obstruction in patients who fail conservative management, particularly those with a prior history of malignancy, radiation exposure, or other risk factors. Diffuse intra-abdominal malignancy may not be readily apparent on preoperative imaging, and definitive diagnosis may only be established at the time of surgical exploration.
